# Innovative virtual reality exposure therapy for anxiety and posttraumatic stress disorder: a meta-analysis of randomised controlled trials

**DOI:** 10.7189/jogh.16.04090

**Published:** 2026-03-27

**Authors:** Yi-Chih Chang, Hidayat Arifin, Tsui-Mei Hung, Chiao-Ling Lin, Jia-You Ye, Li-Chung Pien, Pi-Yu Su, Ruey Chen, Kuei-Ru Chou

**Affiliations:** 1 Department of Nursing, Taipei City Hospital, Songde Branch, Taipei, Taiwan; 2 School of Nursing, College of Nursing, Taipei Medical University, Taipei Taiwan; 3 Department of Medical-Surgical, Emergency, Disaster, and Critical Care Nursing, Faculty of Nursing, Universitas Airlangga, Surabaya, Indonesia; 4 Research Group in Medical-Surgical Nursing, Faculty of Nursing, Universitas Airlangga, Surabaya, Indonesia; 5 Post-Baccalaureate Program in Nursing, College of Nursing, Taipei Medical University, Taipei, Taiwan; 6 Department of Nursing, College of Medicine and Hospital, National Cheng Kung University, Tainan, Taiwan; 7 Psychiatric Research Center, Wan Fang Hospital, Taipei Medical University, Taipei, Taiwan; 8 Department of Nursing, Wan Fang Hospital, Taipei Medical University, Taiwan; 9 Department of Nursing, Taipei Medical University-Shuang Ho Hospital, New Taipei City, Taiwan; 10 Center for Nursing and Healthcare Research in Clinical Practice Application, Wan Fang Hospital, Taipei Medical University, Taipei, Taiwan; 11 Psychiatric Research Center, Taipei Medical University Hospital, Taipei, Taiwan; 12 Neuroscience Research Center, Taipei Medical University, Taipei, Taiwan; 13 Department of Nursing, National Tainan Junior College of Nursing, Tainan, Taiwan

## Abstract

**Background:**

Anxiety disorders impose a substantial burden on individuals and society, affecting mental health, quality of life, and health care systems. Virtual reality exposure therapy (VRET) has emerged as a promising treatment. However, existing meta-analyses often included participants without clinically diagnosed anxiety or related disorders. This study aimed to evaluate the efficacy of VRET in reducing anxiety, phobia, behavioural symptoms, and posttraumatic stress disorder (PTSD) among patients with anxiety-related disorders and PTSD.

**Methods:**

A systematic search was conducted across seven databases – CINAHL, Embase, PubMed, PsycINFO, Web of Science, Cochrane Library, and Medline-OVID. Eligible studies included randomised controlled trials (RCTs) examining the effects of VRET on anxiety-related outcomes. Data were pooled using a random-effects model with Comprehensive Meta-Analysis (version 3.0; Biostat, Englewood, NJ, USA). Effect size were calculated using Hedges’ g, and moderator analyses explored potential factors influencing the effect sizes.

**Results:**

Twenty-six RCTs involving 1649 participants met the inclusion criteria. Overall, VRET demonstrated significant and clinically meaningful effects across multiple domains. It reduced phobia symptoms (Hedges’ g = −0.98; 95% confidence interval (CI) = −1.37, −0.60, *P* < 0.001), increased approach behaviour (Hedges’ g = 0.62; 95% CI = 0.11, 1.12, *P* = 0.02), alleviated anxiety symptoms (Hedges’ g = −0.61; 95% CI = −0.90, −0.33, *P* < 0.001), and mitigated PTSD symptoms (Hedges’ g = −0.51; 95% CI = −0.72, −0.30, *P* < 0.001). Moderator analyses indicated that shorter intervention durations (<60 minutes) were associated with larger treatment effects on anxiety and phobia. VR-based Cognitive Behavioral Therapy (VR-CBT) and VRET demonstrated large and significant effects for phobia.

**Conclusions:**

VRET effectively reduces anxiety, phobia, and PTSD symptoms while enhancing approach behaviour in clinically diagnosed patients. Its immersive and adaptable features make it a valuable exposure-based treatment tool. Further RCTs using advanced VR technologies and standardised protocols are needed to strengthen the evidence and optimise clinical application.

**Registration:**

Open Science Framework (https://doi.org/10.17605/OSF.IO/AD4M2).

The prevalence of anxiety-related disorders, such as panic disorder, social anxiety disorder (SAD), and posttraumatic stress disorder (PTSD), has considerably increased in recent years. At the global level, anxiety disorders affected an estimated 359.2 million individuals in 2021, with an age standardised point prevalence of 4421.9 per 100 000 population, representing an 18% increase since 1990 [1]. In Taiwan, outpatient visits for anxiety, dissociative, stress-related, and somatoform disorders surged by 36% during the same period, highlighting a growing public health concern [2,3]. Posttraumatic stress disorder results from exposure to traumatic events and acute psychological trauma, leading to severe anxiety reactions, intrusive memories, avoidance behaviour, and functional impairments [4,5]. These disorders compromise patients’ mental and physical health, prevent them from engaging in social activities, and diminish their overall quality of life, underscoring a crucial need for effective interventions.

The treatment of anxiety disorders, PTSD, panic disorder, and phobia involves various strategies that can be administered individually or in combination, depending on disease severity and patient requirements [6,7]. Standard treatment approaches for these conditions include personalised psychotherapy, pharmacological interventions, and health-promoting strategies. Psychotherapy includes exposure therapy, cognitive behavioural therapy, and eye movement desensitisation and reprocessing [7–9]. In addition, relaxation techniques such as deep breathing, yoga, and meditation, as well as lifestyle modifications such as a balanced diet, regular exercise, and reduced caffeine and alcohol intake, can help manage anxiety and enhance overall well-being [6,10]. Pharmacological interventions, including antidepressants, are commonly prescribed for symptom management [8].

Advancements in technology have led to the increasing use of virtual reality (VR) as a therapeutic tool. VR exposure therapy (VRET) is an emerging psychological treatment that immerses patients in simulated environments to help them confront and manage anxiety triggers [7,9]. A growing body of research supports its efficacy in treating various mental health conditions [11]. Its unique therapeutic advantages include a safe and controlled exposure environment, customisable and repeatable scenarios for gradual desensitisation, and increased acceptance among patients hesitant about traditional therapies. Additional advantages are real-time physiological feedback, which enhances treatment precision, and technological appeal, which increases patient engagement [12,13].

Previous systematic reviews and meta-analyses have reported that VRET is a clinically effective treatment for various anxiety disorders, specific phobic conditions, SAD, and PTSD [14,15]. However, existing syntheses are limited in several critical respects. Many pooled heterogeneous study designs included non-clinical or subthreshold populations, or combined randomised and nonrandomised evidence, thereby constraining causal inference and clinical interpretability. Moreover, prior reviews largely emphasised symptom reduction alone, without systematically examining behavioural mechanisms (*e.g.* approach and avoidance) or intervention characteristics that may explain variability in treatment response [16]. The present study advances the literature beyond prior meta-analyses in three important ways. First, it synthesises evidence exclusively from randomised controlled trials involving clinically diagnosed anxiety-related disorders, thereby strengthening causal inference and enhancing applicability to clinical practice. Second, it expands the conceptual scope of VRET research by jointly evaluating symptomatic outcomes (anxiety, phobia, PTSD) and clinically meaningful behavioural outcomes (approach and avoidance). Third, through structured subgroup and moderator analyses, this study identifies intervention-level characteristics particularly session duration that may optimise efficacy while minimising adverse effects.

Accordingly, conceptualises anxiety related disorders as sharing common mechanisms of maladaptive fear acquisition, avoidance, and impaired extinction, this meta-analysis aimed to rigorously evaluate the efficacy of VRET across clinically diagnosed anxiety-related disorders. First, this study evaluates the effects of VRET on core symptomatic outcomes including anxiety, phobia, and posttraumatic stress disorder symptoms. Second, evaluate the effects of VRET on clinically relevant behavioural outcomes reflecting fear extinction processes, including approach and avoidance behaviours. Third, it explored whether intervention characteristics and diagnostic subgroups modify treatment effects through predefined subgroup and moderator analyses. In doing so, this study provides novel evidence with direct implications for evidence-based practice, digital mental health implementation, and the scalable delivery of exposure-based interventions.

## METHODS

### Study design

This systematic review and meta-analysis adhered to the Preferred Reporting Items for Systematic Reviews and Meta-Analyses (PRISMA) guidelines [17]. The study protocol was registered with the Open Science Framework (https://doi.org/10.17605/OSF.IO/AD4M2) and was updated prior to reanalysis to prespecify hypotheses regarding treatment effects and potential sources of between-study heterogeneity. Specifically, we hypothesised that virtual reality exposure therapy would improve anxiety, phobia, PTSD, and related behavioural outcomes in clinically diagnosed anxiety-related disorders, and that diagnostic category and intervention session duration would moderate pooled treatment effects. Because this review was conducted using only aggregated, anonymised, and published data, approval from an ethics review board was not required.

### Search strategy and selection criteria

A university librarian was consulted to develop the search strategy and syntaxes. Seven online databases – CINAHL, Embase, PubMed, PsycINFO, Web of Science, Cochrane Library, and Medline-OVID – were comprehensively searched without restrictions on publication status, language, or region. The search was conducted and updated in June 2025. Forward citation mining was performed by identifying articles cited by the included studies, whereas backward citation mining was performed by reviewing the reference lists of previous systematic reviews and other relevant studies. Furthermore, a manual search was performed on Google Scholar to identify additional relevant studies. The search included keywords such as ‘anxiety disorder’ OR ‘phobia’ OR ‘panic’ OR ‘posttraumatic stress disorder’ AND ‘virtual reality’ AND ‘exposure therapy’ (Table S1–2 in the **Online Supplementary Document**).

The inclusion and exclusion criteria were defined using the population, intervention, control, outcomes, and study design framework. Eligible studies included:

1) Participants with anxiety disorders or PTSD, panic disorder, and specific phobias, identified through established diagnostic approaches. These approaches comprised clinician or physician diagnosis based on the Diagnostic and Statistical Manual of Mental Disorders, Fourth Edition (DSM-IV) or Fifth Edition (DSM-5), as well as diagnoses or clinically significant symptoms determined using validated instruments or disorder-specific scales.

2) VRET was operationally defined as an intervention in which immersive virtual environments were used as the primary modality to deliver systematic and repeated exposure to disorder-relevant feared stimuli. To be eligible, exposure delivered through virtual environments had to constitute the core therapeutic component and be administered in a structured and progressive manner, regardless of whether additional therapeutic elements were present. Interventions were included if VRET was delivered as a standalone protocol or embedded within broader treatment packages, such as VR-delivered cognitive behavioural therapy or motion-assisted exposure approaches, provided that exposure remained the principal mechanism of action. Interventions using augmented reality or motion-assisted platforms were included when exposure to feared stimuli occurred through immersive or semi-immersive virtual environments.

3) A control group that received other interventions or standard therapy.

4) Having an RCT design.

The exclusion criteria were as follows:

1) Studies in which VR was used solely for psychoeducation, relaxation training, or non-exposure purposes were excluded being unrelated to the present topic.

2) Studies involving unrelated populations.

3) Non-research articles or studies with an irrelevant design (*e.g.* cross-sectional or case-control).

4) Study protocols.

5) Review articles.

6) Studies offering insufficient data, even after corresponding authors were contacted for missing information.

### Article selection and data extraction

**Figure 1:**
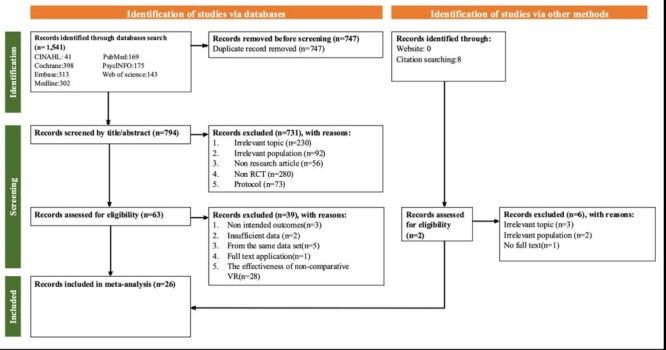
Preferred reporting items for systematic reviews and meta-analyses flowchart.

Two reviewers independently performed the initial article selection and screening, which was followed by the removal of duplicate records. Then, they reviewed the titles and abstracts of the remaining articles to determine eligibility on the basis of the aforementioned inclusion and exclusion criteria. Articles that met the inclusion criteria were subjected to a full-text review to confirm their relevance. Any discrepancies between the reviewers were resolved through discussions within the research team until a consensus was reached, thus ensuring accuracy and reliability (**Figure 1**).

The entire workflow, from article identification to full-text review, was managed using EndNote (version 20.3). To ensure the completeness of the data sets, the corresponding authors of the included studies were contacted for additional information or clarification. Data relevant to the study objective were collected, including authorship, year of publication, study location, and key participant characteristics (*e.g.* demographic characteristics and clinical diagnoses). The extracted data also included information on the interventions, outcome measures, and follow-up durations (**Table 1**).

**Table 1:** Characteristics of the included studies

First author (year), country	Participant characteristics		Interventions		
	**Demographic characteristics**	**Diagnosis**	**Procedure**	**Outcome measurement**	**Follow-up**
Anderson PL, Price M, 2013 [18], USA	Sample size. Overall: 97; EG: 30; CG: 28; mean age = 39.03 ± 11.26 y. sex, n (%): M = 37 (38.1); F = 62 (61.9)	Diagnosis: social anxiety disorder. Diagnostic tool: DSM-IV-TR.	Experiment: VRET. Type of intervention: virtual environments- Therapist: clinical psychologist. Total sessions: 8. Duration: 30 min. Frequency: NI. Total time: 240 min. Control: waitlist	Anxiety: FNE-B	Baseline. Posttest. 3-mo FU. 12-mo F.
Azimisefat P, de Jongh A, 2022 [19], Iran	Sample size. Overall: 30; EG: 15; CG: 15. Mean age: EG = 17.20 ± 0.77 y; CG = 16.93 ± 0.79 y. Sex, n (%): M = 0 (0); F = 30 (100)	Diagnosis: specific phobia. Diagnostic tool: DSM-IV.	Experiment: VRET. Type of intervention: virtual environments. Therapist: clinical psychologist. Total sessions: 6. Duration: 60 min. Frequency: twice a week. Total time: 360 min. Control: waitlist.	Anxiety: ASI-R. Phobia: SMA	Baseline. Posttest
Botella C, Pérez-Ara M,2016 [20], Spain	Sample size. Overall: 63; EG: 32; CG: 31. Mean age (years): Overall: 31.73 ± 10.74; EG: 31.03 ± 10.08; CG: 32.45 ± 11.50. Sex, *n* (%); M: 4 (6.3); F: 59 (93.7)	Diagnosis: specific phobia (animal subtype) to cockroaches or spiders. Subtype: cockroaches Phobia, n (%): Overall: 54 (85.7); EG: 27 (84.4); CG: 27 (87.1). Spider phobia, n (%): Overall: 9 (14.3); EG: 4 (12.9); CG: 5 (15.6). Diagnostic tool: DSM-IV-TR	Experiment: ARET. Type of intervention: head-mounted display. Therapist: clinical psychologist. Total sessions: 1. Duration: 180 min. Frequency: once a week. Total time: 180 min. Control: in vivo exposure	Phobia: FSQ. Avoidance behaviour: BAT	Baseline. Posttest. 6-mo FU
Bourassa KJ, Smolenski DJ,2020 [21], USA	Sample size: Overall: 108; EG: 54; CG: 54. Mean age: EG: 29.5 ± 6.5 y; CG: 30.4 ± 6.5 y. Sex, n (%): M: 52 (96.3); F: 2 (3.7)	Diagnosis: PTSD. Diagnostic tool: CAPS-IV	Experiment: VRET. Type of intervention: head-mounted display. Therapist: NI. Total sessions: 10. Duration: 90–120 min. Frequency: twice a week. Total time: 900–1200 min. Control: waitlist	PTSD: CAPS	Baseline. Midtest. Posttest
Emmelkamp PM, Krijn M,2002 [22], the Netherlands	Sample size: Overall: 33; EG: 17; CG: 16. Mean age: 43.97 ± 9.34 y. Sex, *n* (%): M: 18 (54.55); F: 15 (45.45)	Diagnosis: acrophobia. Diagnostic tool: DSM-IV	Experiment: VRET. Type of intervention: VR head-mounted display. Therapist: clinical psychologist. Total sessions: 3. Duration: 60 min. Frequency: once a week. Total time: 180 min. Control: in vivo exposure	Phobia: AQ. Approach behaviour: BAT	Baseline. Posttest. 6-mo FU
Freeman D, Haselton P, 2018 [23], UK	Sample size: Overall: 100; EG: 49; CG: 51. Mean age: EG: 42.7 ± 17.0 y; CG: 45.7 ± 11.1 y. Sex, n (%): M: 48 (48); F: 52 (52)	Diagnosis: acrophobia. Diagnostic tool: HIQ and DSM-5	Experiment: VRET. Type of intervention: head-mounted display. Therapist: no therapist, automated system. Total sessions: 6. Duration: 30 min. Frequency: six over a period of 2 weeks. Total time: 180 min. Control: usual care	Phobia: AQ	Baseline. Posttest. 2-week FU
Gamito P, Oliveira J, 2010 [24], Portugal	Sample size: Overall: 8; EG: 5; CG: 3. Mean age: 63.5 ± 4.43 y. Sex, n (%): M: 8 (100); F: 0 (0)	Diagnosis: PTSD. Diagnostic tool: CAPS and DSM-IV	Experiment: VRET. Type of intervention: head-mounted display. Therapist: clinical psychologist. Total sessions: 12. Duration: NI. Frequency: NI. Total time: NI. Control: waitlist	PTSD: CAPS	Baseline. Posttest
Garcia-Palacios A, Hoffman H, 2002 [25], Spain	Sample size: Overall: 23; EG: 12; CG: 11. Mean age: 29.25 ± 10.79 y. Sex, n (%): M: 3 (9.1); F: 20 (90.9)	Diagnosis: phobia. Subtype: specific phobia, animal phobia, and spider phobia. Diagnostic tool: DSM-IV	Experiment: VRET. Type of intervention: head-mounted display. Therapist: clinical psychologist. Total sessions: 4. Duration: 60 min. Frequency: once a week. Total time: 240 min. Control: waitlist	Phobia: FSQ. Approach behaviour: BAT	Baseline. Posttest
Gujjar KR, van Wijk A, 2019 [26], Malaysia	Sample size: Overall: 30; EG: 15; CG: 15. Mean age: EG: 25.3 ± 8.6 y; CG: 23 ± 8.9 y. Sex, n (%): M: 12 (40); F: 18 (60)	Diagnosis: dental phobia. Diagnostic tool: MDAS	Experiment: VRET. Type of intervention: head-mounted display. Therapist: clinical psychologist. Total sessions: 1. Duration: 40.06 min. Frequency: once a week. Total time: 40.06 min. Control: informational pamphlet	Anxiety: MDAS. Avoidance behaviour: BAT	Baseline. Pretest. Posttest. 1-week FU. 3-mo FU. 6-mo FU
Jiang MYW, Upton E, 2020 [12], Australia	Sample size: Overall: 43; EG: 21; CG: 22. Mean age: EG:22.38 ± 4.75 y; CC:24.45 ± 7.67 y. Sex, n (%): M: 8 (18.61); F: 35 (81.39)	Diagnosis: phobia. Subtype: blood–injury–injection phobia. Diagnostic tool: DSM-V	Experiment: VRET. Type of intervention: head-mounted display. Therapist: clinical psychologist. Total sessions: 1. Duration: 90 min. Frequency: once a week. Total time: 90 min. Control: waitlist	Anxiety: MDAS. Phobia: MBPI	Baseline. Posttest. 3-mo FU
Kampmann IL, Emmelkamp PM, 2016 [27], the Netherlands	Sample size: Overall: 40; EG: 20; CG: 20. Mean age: EG: 39.65 ± 11.77 y; CG: 33.50 ± 11.44 y. Sex, n (%): M: 17 (42.5); F: 23 (57.5)	Diagnosis: social anxiety disorder. Diagnostic tool: DSM-IV	Experiment: VRET. Type of intervention: head-mounted display. Therapist: clinical psychologist. Total sessions: 10. Duration: 60 min. Frequency: twice a week. Total time: 600 min. Control: waitlist	Anxiety: LSAS-SR	Baseline. Posttest. 3-mo FU
Lacey C, Frampton C, 2023 [28], New Zealand	Sample size: Overall: 126; EG: 63; CG: 63. Mean age: EG: 42.1 ± 12.5 y; CG: 42.5 ± 13.8 y. Sex, n (%): M: 25 (19.8); F: 101 (80.2)	Diagnosis: specific phobias. Diagnostic tool: BSSS	Experiment: VR-CBT. Type of intervention: oVRcome. Therapist: smartphone application (app). Total sessions: 28. Duration: 10. Frequency: 7 times a week. Total time: 280 min. Control: CG: waitlist	Phobia: SMSP	Baseline. Posttest. 3-mo FU
Maltby N, Kirsch I, 2002 [29], USA	Sample size: Overall: 43; EG: 20; CG: 23. Mean age: EG: 22.8 ± 12.6 y; CG: 15 ± 9.5 y. Sex, n (%): M: 9 (21); F: 34 (79)	Diagnosis: specific phobia. Subtype: situational phobia. Diagnostic tool: DSM-IV	Experiment: VRET. Type of intervention: head-mounted display. Therapist: clinical psychologist. Total sessions: 5. Duration: 50 min. Frequency: NI. Total time: 250 min. Control: attention placebo group	Anxiety: FAS	Baseline. Posttest. 6-mo FU
McLay RN, Wood DP, 2011 [30], USA	Sample size: Overall: 20; EG: 10; CG: 10. Mean age: EG: 28 ± NI years; CG: 28.8 ± NI years. Sex, n (%): M: 19 (95); F: 1 (5)	Diagnosis: PTSD. Diagnostic tool: CAPS	Experiment: VRET. Type of intervention: head-mounted display. Therapist: clinical psychologist. Total sessions: 20. Duration: 90 min. Frequency: twice a week. Total time: 1800 min. Control: usual care. (prolonged exposure, cognitive processing therapy, eye movement desensitisation and reprocessing, group therapy, psychiatric medication management, substance rehabilitation, inpatient services, or a combination of these)	PTSD: CAPS	Baseline. Posttest (10 weeks).
McLay RN, Baird A, 2017 [31], USA	Sample size: Overall: 81; EG: 43; CG: 38. Mean age: EG: 33 ± 8.33 y; CG: 32 ± 7.71 y. Sex, n (%): M: 78 (96.30); F: 3 (3.70)	Diagnosis: PTSD. Diagnostic tool: DSM-IV	Experiment: VRET. Type of intervention: head-mounted display. Therapist: clinical psychologist. Total sessions: 18. Duration: 90 min. Frequency: twice a week. Total time: 1620 min. Control: control exposure therapy (still images [no virtual reality], prolonged exposure)	PTSD: CAPS	Baseline; Posttest; 1-week FU; 3-mo FU
Michaliszyn D, Marchand A, 2010 [32], Canada	Sample size: Overall: 32; EG: 16; CG: 16. Mean age: 29.10 ± 7.99 y. Sex, n (%): M: 1 (3.13); F: 31 (96.87)	Diagnosis: phobia. Subtype: spider phobia. Diagnostic tool: DSM-IV, SBQ, and FSQ	Experiment: VRET. Type of intervention: head-mounted display. Therapist: clinical psychologist. Total sessions: 8. Duration: 90 min. Frequency: twice a week. Total time: 720 min. Control: in vivo exposure	Phobia: FSQ-F. Approach behaviour: BAT	Baseline. Midtest. Posttest. 3-mo FU
Miloff A, Lindner P, 2019 [33], Sweden	Sample size: Overall: 100; EG: 50; CG: 50. Mean age: EG: 34.06 ± 10.92 y; EG: 34.04 ± 9.85 y. Sex, n (%): M: 16 (16); F: 83 (83); Other: 1 (1)	Diagnosis: phobia. Subtype: spider phobia. Diagnostic tool: DSM-IV	Experiment: VRET. Type of intervention: head-mounted display. Therapist: no therapist, automated system. Total sessions: 1. Duration: 180 min. Frequency: once a week. Total time: 180 min. Control: in vivo exposure	Anxiety: GAD-7. Phobia: SPQ. Approach behaviour: BAT	Baseline. Posttest. 3-mo FU. 12-mo FU
Ready DJ, Gerardi RJ, 2010 [34], USA	Sample size: Overall: 11; EG: 6; CG: 5. Mean age: EG: 57 ± 3.02 y; CG: 58 ± 3.05 y. Sex, n (%): M: 11 (100); F: 0 (0)	Diagnosis: PTSD. Diagnostic tool: structured clinical interview for DSM-IV	Experiment: VRET. Type of intervention: head-mounted display. Therapist: clinical psychologist. Total sessions: 10. Duration: 90 min. Frequency: NI. Total time: 900 min. Control: present-centred therapy	PTSD: CAPS	Baseline. Posttest. 6-mo FU
Reeves R, Elliott A, 2021 [35], a USA	Sample size: Overall: 26; EG: 17; CG: 9. Mean age: EG: 27.40 ± 9.25 y; CG: 24.17 ± 6.53 y. Sex, n (%): M: 1 (4); F: 25(96)	Diagnosis: anxiety. Subtype: public speaking anxiety. Diagnostic tool: PSAS	Experiment: EG: VRET (360 audience). Type of intervention: head-mounted display. Therapist: clinical psychologist. Total sessions: 4. Duration: 4-15 min. Frequency: twice a week. Total time: 16-60 min. Control: no treatment	Anxiety: LSAS-SR	Baseline. Session 2. Session 3. Posttest. 10-week FU
Reeves R, Elliott A, 2021 [35], b USA	Sample size: Overall: 25; EG: 16; CG: 9. Mean age: EG: 26.06 ± 6.79 y; CG: 24.17 ± 6.53 y. Sex, n (%): M: 2 (8); F: 23 (92)	Diagnosis: anxiety. Subtype: public speaking anxiety. Diagnostic tool: PSAS	Experiment: EG: VRET (360 empty). Type of intervention: head-mounted display. Therapist: clinical psychologist. Total sessions: 4. Duration: 4-15 min. Frequency: twice a week. Total time: 16-60 min. Control: no treatment	Anxiety: LSAS-SR	Baseline. Session 2. Session 3. Posttest. 10-week FU
Reger GM, Koenen-Woods P, 2016 [36], USA	Sample size: Overall: 108; EG: 54; CG: 54. Mean age: EG: 29.52 ± 6.47 y; CG: 30.39 ± 6.45 y. Sex, n (%): M: 105 (97.2); F: 3 (2.8)	Diagnosis: PTSD. Diagnostic tool: DSM-IV	Experiment 1: VRET. Type of intervention: head-mounted display. Therapist: clinical psychologist. Total sessions: 10. Duration: 90-120min. Frequency: once or twice a week. Total time: 900-1200min. Control: waitlist	Anxiety: BAI. PTSD: CAPS	Baseline. Midtest. Posttest. 3-mo FU. 26-week FU
Repetto C, Gaggioli A, 2013 [37], Italy	Sample size: Overall: 16; EG: 8; CG: 8. Mean age: EG: 48.5 ± 12.66 y; CG: 49.25 ± 9.85 y. Sex, n (%): NI	Diagnosis: anxiety. Subtype: generalized anxiety disorder. Diagnostic tool: DSM-IV	Experiment: VRET-CBT. Type of intervention: head-mounted display. Therapist: clinical psychologist. Total sessions: 8. Duration: 90 min. Frequency: twice a week. Total time: 720 min. Control: waitlist	Anxiety: BAI	Baseline. Posttest
Rothbaum BO, Hodges LF, 1995 [38], USA	Sample size: Overall: 20; EG: 12; CG: 8. Mean age: 20 ± 4.00 y. Sex, n (%): M: 12 (60); F: 8 (40)	Diagnosis: phobia. Subtype: acrophobia. Diagnostic tool: AQ	Experiment: VRET. Type of intervention: head-mounted display. Therapist: clinical psychology student. Total sessions: 7. Duration: 35–45 min. Frequency: once a week. Total time: 245–315 min. Control: no treatment	Phobia: ATHQ	Baseline. Posttest. 2-mo FU
Rothbaum BO, Anderson P, 2006 [39], USA	Sample size: Overall: 54; EG: 29; CG1: 25. Mean age: EG: 38.62 ± 9.16 y; CG: 37.20 ± 9.5 y. Sex, n (%): M: 9 (16.67); F: 45 (83.33)	Diagnosis: phobia. Subtype: fear of flying and. Acrophobia. Diagnostic tool: DSM-IV	Experiment 1: VRET. Type of intervention: head-mounted display. Therapist: clinical psychologist. Total sessions: 8. Duration: 90 min. Frequency: once or twice a week. Total time: 720 min. Control: waitlist	Phobia: QAF	Baseline. Posttest. 6-mo FU. 12-mo FU
Rus-Calafell M, Gutiérrez-Maldonado J, 2013 [40], Spain	Sample size: Overall: 15; EG: 7; CG: 8. Mean age: EG: 37.14 ± 14.28 y; CG: 36.13 ± 12.59 y. Sex, n (%): M: 2 (13.33); F: 13 (85.7)	Diagnosis: phobia. Subtype: fear of flying. Diagnostic tool: DSM-IV	Experiment: VRET. Type of intervention: head-mounted display. Therapist: clinical psychologist. Total sessions: 6. Duration: 60-75 min. Frequency: twice a week. Total time: 360-450 min. Control: imaginal exposure	Anxiety: FFS	Baseline. Posttest. After real flight. 6-mo FU
van Gelderen MJ, Nijdam MJ, 2020 [41], the Netherlands	Sample size: Overall: 43; EG: 22; CG: 21. Mean age: 42.18 ± 9.36 y. Sex, n (%): M: 42 (97.70); F: 1 (2.30)	Diagnosis: PTSD. Subtype: NI. Diagnostic tool: DSM-V	Experiment: 3MDR (VR-CBT). Type of intervention: virtual environments. Therapist: clinical psychologist. Total sessions: 6. Duration: 70–90 min. Frequency: once a week. Total time: 420–540 min. Control: nonspecific treatment	PTSD: CAPS-5. Avoidance behaviour: PABQ	Baseline. 6-week FU. 12-week FU. 16-week FU (primary end point)
Zainal NH, Chan WW, 2021 [42], USA	Sample size: Overall: 44; EG: 26; CG: 18. Mean age: 23.30 ± 9.32 y. Sex, *n* (%): M: 10 (22.70); F: 34 (77.30)	Diagnosis: social anxiety disorder. Diagnostic tool: DSM-5	Experiment: VR-CBT. Type of intervention: head-mounted display. Therapist: no therapist, automated system. Total sessions: 4. Duration: 50–60 min. Frequency: twice a week. Total time: 100–120 min. Control: waitlist	Anxiety: SIAS	Baseline. Posttest. 3-mo FU. 6-mo FU

AQ – Acrophobia Questionnaire, ARET – augmented reality exposure therapy, ATHQ – Attitude Towards Heights Questionnaire, BAI – Beck Anxiety Inventory, BAT – Behavioral Avoidance Test, BSSS – Brief Standard Self-rating Scale, CAPS – Clinician-Administered PTSD Scale, CG – control group, DSM-IV – Diagnostic and Statistical Manual of Mental Disorders, Fourth Edition, DSM-IV-TR – Diagnostic and Statistical Manual of Mental Disorders, Fourth Edition, Text Revision, EG – experimental group, F – female, FAS – Flight Anxiety Situations Questionnaire, FFS – Fear of Flying Scale, FNE-B – Fear of Negative Evaluation–Brief Form, FSQ-F – French version of the Fear of Spiders Questionnaire, FSQ – Fear of Spiders Questionnaire, FU – follow-up, GAD-7 – 7-item Generalized Anxiety Disorder scale, HIQ – Heights Interpretation Questionnaire, LSAS-SR – Liebowitz Social Anxiety Scale–Self-Report, M – male, MBPI – Multidimensional Blood Phobia Inventory, MDAS – Modified Dental Anxiety scale, NI – no information, PABQ – Posttraumatic Avoidance Behaviour Questionnaire, PSAS –Public Speaking Anxiety Scale, PTSD – posttraumatic stress disorder, QAF – Questionnaire on Attitudes Toward Flying, SBQ – Spider Phobia Beliefs Questionnaire, SIAS – Social Interaction Anxiety Scale, SMA – Severity Measure for Acrophobia, SMSP – Severity Measures for Specific Phobia, SPQ – Spider Phobia Questionnaire, VRET – virtual reality exposure therapy

### Measurements

The primary outcome was anxiety, whereas the secondary outcomes were phobia, approach behaviour, avoidance behaviour, and PTSD. Standardised and validated instruments were used to assess all outcomes through structured questionnaires, behavioural tasks, and clinician-administered scales. Anxiety was comprehensively measured using multiple validated instruments assessing both general and specific forms of anxiety. The Beck Anxiety Inventory was used to measure the severity of general anxiety symptoms [17]. The 7-item Generalized Anxiety Disorder scale was used to detect generalised anxiety disorder [43]. Social anxiety was measured using the Liebowitz Social Anxiety Scale-Self-Report [44] and the Social Interaction Anxiety Scale [45]. Flight-related anxiety was measured using the Flight Anxiety Situations questionnaire [46] and the Fear of Flying Scale [47]. Dental anxiety was measured using the Modified Dental Anxiety Scale [48]. Anxiety related to social evaluation and fear of judgment was measured using the Fear of Negative Evaluation-Brief scale [49].

Phobia-related outcomes were measured using instruments assessing specific phobic stimuli. The Acrophobia Questionnaire [50] and the Attitude Towards Heights Questionnaire [51] were used to measure fear of heights. The Spider Phobia Questionnaire [52] and the Fear of Spiders Questionnaire [52] were used to measure arachnophobia. The Multidimensional Blood Phobia Inventory was used to measure blood-injection-injury phobia [53]. The Questionnaire on Attitudes Toward Flying [54] was used to measure flying-related attitudes and fear. Approach behavioural were measured using the Behavioral Approach Test [55], which objectively assesses participants’ ability and willingness to engage with feared stimuli or situations, offering insights into adaptive coping mechanisms and avoidance reduction. Avoidance tendencies were explored using two validated instruments. The Behavioral Avoidance Test was used to evaluate the degree of avoidance in feared situations, indicating the severity of avoidance behaviour. The Posttraumatic Avoidance Behaviour Questionnaire [56] was specifically used to assess avoidance behaviour related to traumatic experiences, revealing patterns that likely contribute to the persistence of PTSD symptoms. Posttraumatic stress disorder symptoms were assessed using the Clinician-Administered PTSD Scale (CAPS) [57] – the gold standard for PTSD diagnosis. The CAPS facilitates a detailed evaluation of symptom severity, incorporating clinician-rated symptom frequency and intensity, aligned with relevant diagnostic criteria outlined in the Diagnostic and Statistical Manual of Mental Disorders.

### Quality assessment

Two reviewers independently evaluated the quality of all included RCTs by using the revised Cochrane Risk of Bias tool (version 2.0) [58]. Potential biases were systematically examined across five key domains: bias arising from the randomisation process, bias arising from deviations from intended interventions, bias resulting from missing outcome data, bias in outcome measurement, and bias in the selection of reported results. Each domain was rigorously examined to ensure a comprehensive assessment of study validity.

Between-reviewer discrepancies were resolved through discussions with an expert reviewer, ensuring the reliability of the quality assessment process. In accordance with the guidelines outlined in the Cochrane Handbook, the overall risk of bias for each study was classified as high risk, some concerns, or low risk. This classification facilitated a nuanced understanding of the methodological rigor and reliability of the included studies.

### Statistical analysis

The meta-analysis was performed using Comprehensive Meta-Analysis (CMA; version 3.0), a robust platform designed for advanced statistical analysis in research synthesis. All of the included studies were RCTs. Because the outcome measures were continuous variables that varied across the included studies (because of the use of different measurement tools and units), effect sizes were standardised to a common metric, ensuring meaningful comparisons and consistency in data interpretation.

To address heterogeneity inherent to transdiagnostic synthesis, this meta-analysis implemented multiple design and analytic strategies. Analyses were restricted to randomised controlled trials with clinically confirmed diagnoses to enhance internal validity. Hedges’ *g* was used to synthesise outcomes measured with different instruments while maintaining comparability across disorders. Random effects models were applied to account for between study variability in mechanisms, symptom profiles, and expected effect sizes. In addition, diagnostic categories and intervention characteristics were examined using predefined subgroup and moderator analyses to evaluate sources of heterogeneity and support transparent interpretation of pooled estimates.

Effect sizes were calculated as standardised mean differences using Hedges’ g. Post-treatment between-group differences were preferentially extracted; when unavailable, change-from-baseline scores were used, followed by pre-post within-group data when necessary. All effect sizes and variance estimates were computed using CMA 3.0, which applies appropriate statistical formulas for post-test differences, change scores, and pre-post data without requiring imputation of pre-post correlation coefficients. A predefined hierarchy was applied to ensure consistency and avoid mixing incompatible effect size metrics. To prevent unit-of-analysis errors, only one effect size per study per outcome domain was included. When multiple measures were reported, selection prioritised clinician-administered instruments or the most widely used and validated scale, and post-intervention outcomes closest to treatment completion were used for the primary analysis. Notably, these effect sizes were interpreted following the guidelines of Leppink, O'Sullivan and Winston [59], who considered *g* values of 0.2, 0.5, and 0.8 to be indicative of small, medium, and large effect sizes, respectively. This approach ensured a consistent framework for assessing the strength and clinical significance of intervention effects across the included RCTs.

Several included trials involved more than two intervention or control arms, which may introduce duplication bias if not handled appropriately. To prevent unit-of-analysis errors, predefined procedures were applied [58]. When multiple intervention arms were compared with a single control group and were conceptually similar, intervention arms were combined by summing sample sizes and calculating pooled means and standard deviations so that each participant contributed only once to the analysis. When intervention arms were distinct and required separate comparisons, the shared control group was divided proportionally across comparisons by splitting the sample size while retaining the original mean and standard deviation. In studies with one intervention arm and multiple control groups, the intervention group sample size was similarly split. When additional arms were not relevant to the meta-analysis objectives, only the eligible comparison was retained. Across all analyses, only one effect size per study per outcome domain was included, ensuring independence of observations and minimising the risk of duplicated samples.

Heterogeneity among the included studies was evaluated using the χ^2^ test (Cochrane’s *Q* test), which examines whether observed variations in effect sizes exceed those expected by chance alone. A significant *Q* statistic (*P* < 0.1) was interpreted as evidence of heterogeneity. The *I*^2^ statistic was calculated to quantify the degree of heterogeneity, with thresholds of 25%, 50%, and 75% indicating low, moderate, and high heterogeneity, respectively. When heterogeneity was detected, a random-effects model was used to account for within- and between-study variability. When substantial heterogeneity was present, subgroup analyses and meta regression were conducted to explore potential sources, including diagnostic category, intervention characteristics, and outcome domains.

To assess potential publication bias, funnel plots were generated when a minimum of 10 studies were available, in accordance with established methodological recommendations. Visual inspection of funnel plot asymmetry was used to identify potential publication bias or small-study effects. In addition, forest plots were generated to display pooled effect sizes of the included studies with their corresponding 95% CIs, providing a summary of the overall findings of the meta-analysis.

## RESULTS

### Study description

The literature search returned 1541 articles. All retrieved references were imported into EndNote (version 20.3) for management and deduplication, which led to the removal of 747 duplicate articles. The remaining 794 articles were rigorously screened on the basis of the inclusion and exclusion criteria. Titles and abstracts were manually reviewed, and 731 articles that did not meet the eligibility criteria were excluded. Subsequently, 63 articles were subjected to a full-text review, during which 39 articles were excluded for reasons such as insufficient data, irrelevance to the research question, or failure to meet methodological standards outlined in the study protocol. A manual search of the reference lists of the included studies revealed one additional eligible article. Ultimately, 26 articles meeting the eligibility criteria were included in the final meta-analysis. A PRISMA flowchart depicting the detailed selection process is presented in **Figure 1**.

### Study characteristics

All 26 articles were published between 1995 and 2023 and involved patients diagnosed with anxiety disorders such as SAD (n = 3), specific phobic conditions (n = 10), acrophobia (n = 3), PTSD (n = 7), dental phobia (n = 1), public speaking anxiety (n = 1), or generalised anxiety disorders (n = 1). These studies were conducted in the USA (n = 11), Spain (n = 3), the Netherlands (n = 3), the UK (n = 1), Portugal (n = 1), Malaysia (n = 1), Australia (n = 1), Sweden (n = 1), Italy (n = 1), Iran (n = 1), New Zealand (n = 1), and Canada (n = 1). Virtual Reality Exposure Therapy was administered using head-mounted displays in 22 studies and in alternative virtual environments in four studies. The number of therapeutic sessions ranged from 1 to 14, whereas the duration ranged from approximately 40 to 180-minute each. Most of the interventions were delivered by clinical psychologists (n = 21), with a few being delivered through automated systems without therapist involvement (n = 4). The studies varied in terms of control conditions: waitlist, 12 studies; in vivo exposure, four studies; usual care, two studies; informational pamphlets, one study; attention placebo group, one study; and other forms of exposure therapy, four studies. Outcomes were primarily measured using standardised anxiety and phobia scales such as the Fear of Spiders Questionnaire, Liebowitz Social Anxiety Scale-Self-Report, CAPS, and Behavioral Approach Test. Follow-up periods ranged from one week to one year.

### Results of quality assessment

Among the 26 studies that implemented intention-to-treat analysis, 70.0% had a low risk of bias, 25.0% had some concerns, and 5% had a high risk of bias (Figure S1 in the **Online Supplementary Document**).

### Effect of VRET on anxiety

**Figure 2:**
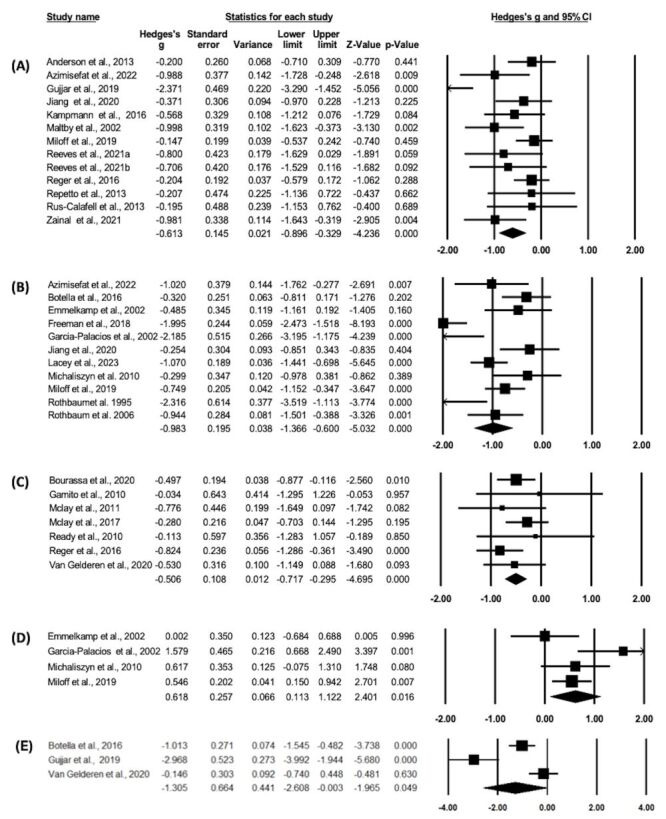
The effect of virtual reality exposure therapy: **Panel A.** Anxiety. **Panel B.** Phobia. **Panel C.** Posttraumatic stress disorder. **Panel D.** Approach behaviour. **Panel E.** Avoidance behaviour.

Thirteen studies [12,18,19,26,27,29,33,35–37,40,42] were included in the meta-analysis evaluating the efficacy of VRET in reducing anxiety. Pooled analysis revealed a moderate effect size (Hedges’ *g* = −0.61; 95% CI = −0.90, −0.33, *P* < 0.001). Heterogeneity analysis indicated moderate heterogeneity among the included studies (*Q* = 30.66; df(Q) = 12, *I*^2^ = 60.87%, *P* = 0.002) (**Figure 2**, Panel A). To ensure the robustness of the findings, we conducted a sensitivity analysis by systematically excluding one study at a time. The results remained uniform, with the effect size unchanged (Hedges’ *g* = −0.61; 95% CI = −0.90, −0.33), indicating that the primary combined effect size was not disproportionately influenced by any single study (Table S3 in the **Online Supplementary Document**).

Publication bias was assessed using both visual and statistical methods. The funnel plot revealed asymmetry, with fewer studies appearing on the left side than on the right side, highlighting the potential presence of publication bias. This observation was supported by the results of Egger’s regression intercept test, which yielded a *P*-value of 0.025, further confirming the likelihood of bias. To address the potential bias, we adjusted for missing studies by using Duval and Tweedie’s trim-and-fill method. The results indicated that the effect size was −0.71 after adjustment. Because the adjusted 95% CI value (−0.996, −0.42) suggested no significant change from the original pooled effect size, publication bias was assumed to have a minimal effect on the overall results, underscoring the robustness of our conclusions (Table S4 in the **Online Supplementary Document**).

### Effect of VRET on phobia

Eleven studies [12,19,20,22,23,25,28,32,33,38,39] were included in the meta-analysis evaluating the efficacy of VRET in reducing phobia. Pooled analysis revealed a large effect size (Hedges’ *g* = −0.98; 95% CI = −1.37, −0.60, *P* < 0.001), indicating that VRET can effectively mitigate phobic symptoms. However, the heterogeneity among the included studies was high (*Q* = 47.39; df(Q) = 10, *I*^2^ = 78.90%, *P* < 0.001), suggesting significant variability in the results (**Figure 2**, Panel B). Sensitivity analysis confirmed the robustness of the results. The effect size remained uniform (Hedges’ *g* = −1.008; 95% CI = −1.40, −0.61), implying that the primary findings were stable and not unduly influenced by any single study (Table S3 in the **Online Supplementary Document**). Notably, excluding the study by Rothbaum et al. (1995), which had a high risk of bias, did not alter the effect size (Hedges’ *g* = −0.92; 95% CI = −1.30, −0.53), further supporting the robustness of the findings.

Publication bias was assessed using both visual and statistical methods. The funnel plot exhibited rough symmetry on both sides, suggesting no substantial evidence of bias. This finding was corroborated by the results of Egger’s test, which yielded a *P*-value of 0.60, confirming the absence of publication bias (Table S4 in the **Online Supplementary Document**).

### Effect of VRET on PTSD

Seven studies [21,24,30,31,34,36,41] were included in the meta-analysis evaluating the efficacy of VRET in mitigating symptoms of PTSD. Pooled analysis revealed a moderate and significant effect size (Hedges’ *g* = −0.51; 95% CI = −0.72, −0.30, *P* < 0.001), indicating that VRET effectively ameliorated PTSD symptoms. Furthermore, heterogeneity analysis suggested low variability across the included studies (*Q* = 4.26; df(Q) = 6, *I*^2^<0.001%, *P* = 0.64), implying a high degree of consistency in the results (**Figure 2**, Panel C). Sensitivity analysis confirmed the robustness of the pooled effect size. The effect size remained uniform (Hedges’ *g* = −0.52; 95% CI = −0.73, −0.30), confirming the reliability of the primary findings (Table S3 in the **Online Supplementary Document**).

### Effect of VRET on approach behaviour

Four studies [22,25,32,33] were included in the meta-analysis evaluating the efficacy of VRET in enhancing approach behaviour. Pooled analysis revealed a moderate effect size (Hedges’ *g* = 0.62; 95% CI = 0.11, 1.12, *P* = 0.02), suggesting that VRET exerted a significant and moderate effect on approach behaviour. However, heterogeneity analysis indicated a high degree of variability across the included studies (*Q* = 7.38; df(Q) = 3, *I*^2^ = 59.34%, *P* = 0.06), highlighting significant differences in the results (**Figure 2**, Panel D). Sensitivity analysis confirmed the stability of the findings. The effect size remained uniform (Hedges’ *g* = 0.63; 95% CI = 0.11, 1.15), confirming that the overall results were robust and not disproportionately influenced by any single study (Table S3 in the **Online Supplementary Document**).

### Effect of VRET on avoidance behaviour

Three studies [20,26,41] were included in the meta-analysis evaluating the efficacy of VRET in mitigating avoidance behaviour. Pooled analysis revealed a large effect size (Hedges’ *g* = −1.31; 95% CI = −2.61, −0.01, *P* = 0.05), indicating that VRET exerted a significant and large effect on avoidance behaviour. However, heterogeneity analysis indicated high variability across the included studies (*Q* = 21.99; df(Q) = 2, *I*^2^ = 90.91%, *P* < 0.001), highlighting significant differences in the results (**Figure 2**, Panel E). Sensitivity analysis confirmed the stability of the findings. The effect size remained uniform (Hedges’ *g* = −1.34; 95% CI = −2.67, −0.01), confirming that the primary results were robust and not unduly influenced by any single study (Table S3 in the **Online Supplementary Document**).

### Results of moderator analysis

Subgroup analyses were conducted to examine potential moderators of treatment effects for anxiety outcomes. Regarding intervention format, studies using active controls showed a large but non-significant pooled effect (Hedges’ g = −0.89; 95% CI = −1.80, 0.02, *P* = 0.054), whereas studies using passive controls demonstrated a moderate and statistically significant effect (Hedges’ g = −0.47; 95% CI = −0.68, −0.26, *P* < 0.001). However, the subgroup difference was not statistically significant (Q = 0.79, *P* = 0.373), indicating that control type did not significantly moderate treatment effects. In terms of intervention type, VRET yielded a significant reduction in anxiety symptoms (Hedges’ g = −0.61; 95% CI = −0.92, −0.29, *P* < 0.001), while VR-CBT showed a large but non-significant effect (Hedges’ g = −0.66; 95% CI = −1.41, 0.08, *P* = 0.080). The subgroup comparison was not significant (Q = 0.01, *P* = 0.892), suggesting no differential effect between VRET and VR-CBT. Intervention duration significantly moderated treatment effects. Sessions lasting ≤60 minutes produced a large and statistically significant effect (Hedges’ g = −0.87; 95% CI = −1.30, −0.44, *P* < 0.001), whereas longer sessions (>60 minutes) resulted in a smaller but significant effect (Hedges’ g = −0.25; 95% CI = −0.47, −0.02, *P* = 0.027). The subgroup difference was statistically significant (Q = 6.39, *P* = 0.011), indicating that shorter interventions were associated with greater anxiety reduction. For diagnostic categories, significant effects were observed for anxiety disorders (Hedges’ g = −0.54; 95% CI = −0.82, −0.25, *P* < 0.001) and phobia (Hedges’ g = −0.80; 95% CI = −1.38, −0.22. *P* = 0.007), while PTSD showed a non-significant effect (Hedges’ g = −0.20; 95% CI = −0.57, 0.17, *P* = 0.288). No significant subgroup difference was detected across diagnostic categories (Q = 3.40, *P* = 0.183) (**Table 2**). Meta-regression analysis revealed no significant correlations between anxiety outcomes and the following variables (**Table 3**).

**Table 2:** Results of subgroup analysis

Variables	Number of studies	Hedges’ *g* (95% confidence interval)	Null hypothesis test (two-tailed)		Homogeneity test	
			***Z***	*P-*value*	***Q***	*P-*value*
**Anxiety**						
Intervention format						
*Active control*	4	−0.89 (−1.80, 0.02)	−1.93	0.054	0.79	0.373
*Passive control*	9	−0.47 (−0.68, −0.26)	−4.30	<0.001		
Type of Intervention						
*VRET*	11	−0.61 (−0.92, −0.29)	−3.81	<0.001	0.01	0.892
*VR-CBT*	2	−0.66 (−1.41, 0.08)	−1.75	0.080		
Duration (min)						
*≤60*	8	−0.87 (−1.30, −0.44)	−4.00	<.0001	6.39	0.011
*>60*	5	−.25 (−0.47, −0.02)	−2.21	<0.027		
Diagnosis						
*Anxiety*	6	−0.54 (−0.82, −0.25)	−3.76	<0.001	3.40	0.183
*Phobia*	6	−0.80 (−1.38, −0.22)	−2.70	0.007		
*Posttraumatic stress disorder*	1	−0.20 (−0.57, 0.17)	−1.06	0.288		
**Phobia**						
Intervention format						
*Active control*	5	−0.78 (−1.43, −0.13)	−2.37	<0.001	0.75	0.384
*Passive control*	6	−1.14 (−1.63, −0.66)	−4.61	<0.001		
Type of Intervention						
*ARET*	1	−0.32 (−0.81, 0.17)	−1.28	0.020	6.59	0.037
*VR-CBT*	1	−1.07 (−1.44, −0.70)	−5.65	<0.001		
*VRET*	9	−1.07 (−1.55, −0.59)	−4.38	<0.001		
Duration (min)						
*≤60*	6	−1.43 (−1.98, −0.87)	−5.06	<0.001	7.92	0.005
*>60*	5	−0.54 (−0.81, −0.27)	−3.99	<0.001		
Diagnosis						
*Acrophobia*	4	−1.37 (−2.18, −0.57)	−3.34	0.001	5.20	0.074
*Blood–injection–injury phobia*	1	−0.25 (−0.85, 0.34)	−0.83	0.404		
*Specific phobia*	6	−0.77 (−1.07, −0.46)	−4.91	<0.001		

ARET – augmented reality exposure therapy, VR-CBT – VR-based Cognitive Behavioral Therapy, VRET – virtual reality exposure therapy

*Significance was set at *P* < 0.05.

**Table 3:** Results of meta-regression analysis

Variables	Number of studies	Coefficient	Standard error	95% confidence interval	*Z*	*P-*value
Anxiety						
*Mean age (years)*	13	0.02	0.01	−0.01, 0.06	1.41	0.15
*Year of implementation*	13	−0.01	0.03	−0.07, 0.05	−0.35	0.73
*Total number of sessions*	13	0.06	0.04	−0.03, 0.15	1.23	0.22
Phobia						
*Mean age (years)*	11	−0.01	0.02	−0.05, 0.03	−0.32	0.75
*Year of implementation*	11	0.02	0.02	−0.02, 0.07	1.02	0.31
*Total number of sessions*	11	−0.11	0.07	−0.24, 0.03	−1.58	0.11

For phobia outcomes, intervention format did not significantly moderate treatment effects. Studies comparing VR interventions with active controls showed a significant pooled effect (Hedges’ g = −0.78; 95% CI = −1.43, −0.13, *P* < 0.001), while those using passive controls showed an even larger significant effect (Hedges’ g = −1.14; 95% CI = −1.63, −0.66, *P* < 0.001). The subgroup difference was not significant (Q = 0.75, *P* = 0.384). Intervention type significantly moderated phobia outcomes. ARET showed a modest effect (Hedges’ g = −0.32; 95% CI = −0.81, 0.17, *P* = 0.020), whereas VR-CBT (Hedges’ g = −1.07; 95% CI = −1.44, −0.70, *P* < 0.001) and VRET (Hedges’ g = −1.07; 95% CI = −1.55, −0.59, *P* < 0.001) demonstrated large and significant effects. The subgroup difference was statistically significant (Q = 6.59, *P* = 0.037), indicating that intervention type influenced treatment efficacy. Sessions ≤60 minutes resulted in a very large and significant reduction in phobia symptoms (Hedges’ g = −1.43; 95% CI = −1.98, −0.87, *P* < 0.001), whereas longer sessions produced a moderate effect (Hedges’ g = −0.54; 95% CI = −0.81, −0.27, *P* < 0.001). The subgroup difference was significant (Q = 7.92, *P* = 0.005). Across diagnostic categories, significant effects were found for acrophobia (Hedges’ g = −1.37; 95% CI = −2.18, −0.57, *P* = 0.001) and specific phobia (Hedges’ g: −0.77; 95% CI = −1.07, −0.46, *P* < 0.001), while blood-injection-injury phobia showed no significant effect (Hedges’ g = −0.25; 95% CI = −0.85, 0.34, *P* = 0.404). The subgroup difference did not reach statistical significance (Q = 5.20, *P* = 0.074) (**Table 2**). Meta-regression analysis revealed no significant correlations between phobia outcomes and the following variables (**Table 3**).

## DISCUSSION

To our knowledge, this meta-analysis provides a comprehensive quantitative synthesis of the therapeutic effects of VRET. The pooled evidence indicates that VRET significantly reduces phobic symptoms, increases approach behaviour, alleviates anxiety symptoms, and mitigates PTSD-related distress. Exploratory moderator analyses further demonstrated that intervention characteristics contributed to variability in treatment efficacy. Specifically, session durations of ≤60 minutes were associated with significantly larger pooled effects for both anxiety and phobia outcomes, suggesting that brief and focused exposure sessions may optimize therapeutic engagement, sustain attention and presence, and facilitate efficient fear extinction. Moreover, both VR-CBT and VRET yielded large and statistically significant effects in reducing phobia, underscoring the robustness of exposure-based interventions delivered through immersive virtual platforms. These effects likely reflect the capacity of virtual environments to provide highly controlled, repeatable, and individualised exposure scenarios which, when integrated with cognitive restructuring and coping strategies in VR-CBT, promote corrective learning and modification of maladaptive threat appraisals. Collectively, these findings provide empirical support for integrating VRET into clinical practice as an effective and scalable transdiagnostic intervention for anxiety-related disorders.

We examined the effect of VRET on phobic symptoms in individuals with anxiety-related disorders. Although evidence of publication bias was detected, the pooled effect remained stable after adjustment using the trim-and-fill method, indicating that the observed findings were robust and unlikely to be substantially influenced by missing studies. The magnitude of the pooled effect suggests that VRET produces clinically meaningful reductions in phobia severity among individuals with anxiety-related disorders. These findings are consistent with those reported by Carl E, Stein AT, Levihn-Coon A, Pogue JR, Rothbaum B, Emmelkamp Pet al. [60], who likewise observed large effect sizes for VRET in the treatment of phobic disorders. The therapeutic mechanism underlying VRET-mediated reductions in phobia may be attributed to principles of systematic desensitisation and exposure-based learning. Immersive virtual environments enable individuals to confront feared stimuli gradually and repeatedly within a safe, controllable, and predictable setting, thereby facilitating habituation to fear cues and extinction of maladaptive fear responses [61,62]. Moreover, the high degree of presence and realism afforded by virtual environments may enhance emotional engagement and corrective learning, promoting durable modification of threat-related cognitions and avoidance behaviours. Collectively, these mechanisms provide a plausible explanation for the substantial treatment effects observed and further support the clinical utility of VRET for phobia-focused interventions.

VRET was found to significantly increase approach behaviour in individuals with anxiety-related disorders. This finding aligns with the results reported by Morina N, Ijntema H, Meyerbröker K and Emmelkamp PM [63], supporting the premise that virtual environments can effectively facilitate behavioural engagement with feared stimuli. A plausible explanation lies in the immersive and interactive characteristics of virtual reality, which allow individuals to experience graded exposure to anxiety-provoking situations while maintaining a strong sense of presence within a controlled and safe setting. This heightened sense of presence is thought to enhance emotional activation and fear extinction processes, thereby reducing conditioned fear responses and avoidance tendencies [64,65]. Furthermore, VR environments permit repeated, standardised, and therapist-guided exposure that may overcome practical and ethical limitations of in vivo exposure, improving treatment adherence and tolerability. By systematically reducing avoidance behaviour and promoting adaptive coping responses, VRET can strengthen exposure learning and ultimately enhance overall treatment efficacy [66]. These mechanisms highlight the potential of VRET as a powerful tool for modifying maladaptive behavioural patterns central to anxiety-related disorders.

Virtual Reality Exposure Therapy significantly alleviates anxiety symptoms in individuals with anxiety-related disorders. After adjustment for publication bias, the findings continued to demonstrate clinically meaningful effect sizes, indicating the robustness of the treatment effect. The pooled analysis revealed a moderate effect size, confirming that VRET produces a substantial reduction in anxiety symptoms. This result is consistent with the findings of Parsons TD and Rizzo AA [67], who likewise reported a moderate therapeutic effect of VRET on anxiety outcomes. One plausible explanation is that VR-based scenarios can be individualized to match patients’ specific fear hierarchies and therapeutic goals, thereby enhancing engagement, motivation, and adherence to treatment [68]. Moreover, the immersive nature of VR allows patients to confront anxiety-provoking stimuli in a realistic yet controllable environment, facilitating emotional processing and fear extinction. Taken together, these findings suggest that VRET represents a viable alternative to conventional exposure-based therapies for generalised anxiety and related disorders, offering an innovative, adaptable, and potentially more accessible therapeutic approach.

Our study revealed a smaller effect size for the impact of VRET on PTSD outcomes compared with those reported by Deng et al. [69] and Eshuis et al. [14]. This discrepancy may be attributed to heterogeneity in study methodologies, baseline PTSD severity, intervention protocols, and outcome measurement instruments across the included trials and previous investigations [70]. Differences in trauma type, chronicity of symptoms, and duration of intervention may have further contributed to variability in treatment response. Despite the comparatively smaller pooled effect, VRET remains a promising therapeutic modality for PTSD, as it enables structured and gradual exposure to trauma-related cues within a safe and controllable virtual environment, thereby enhancing patient engagement and treatment adherence [68]. In addition, VRET may facilitate the recontextualisation of traumatic memories by allowing repeated exposure under therapist guidance, which can reduce emotional reactivity and support adaptive posttraumatic processing [71]. Collectively, these mechanisms underscore the potential of VRET as an innovative and scalable intervention for PTSD, particularly in settings where traditional in vivo exposure is difficult to implement.

Exploratory moderator analyses in this meta-analysis suggested that VRET sessions lasting ≤ 60 minutes were associated with larger pooled effects for anxiety and phobia outcomes. Because moderator analyses were conducted to explore heterogeneity and were not adjusted for multiplicity, these findings should be interpreted as hypothesis-generating rather than confirmatory. Although adverse events were not systematically evaluated in the present meta-analysis, previous virtual reality implementation studies have reported that session durations of approximately 20–60 minutes are commonly used in clinical VRET protocols and are considered feasible within routine therapeutic settings [72,73]. In addition, early clinical trials have employed brief exposure sessions ranging from 25 to 30 minutes [38], demonstrating that effective exposure-based interventions can be delivered within relatively short session formats. Taken together, while our quantitative synthesis supports the effectiveness of VRET across anxiety-related disorders, further randomised controlled trials directly comparing different session durations and incorporating standardised reporting of tolerability outcomes are required to determine optimal intervention length.

The interpretation of these findings should be considered in light of the geographic distribution of the included studies. Most randomised controlled trials evaluating virtual reality exposure therapy have been conducted in high-income countries, where access to advanced technology, stable digital infrastructure, and trained mental health professionals is more readily available. This concentration reflects the current implementation landscape of VRET rather than selective study inclusion. As a result, the pooled estimates predominantly represent treatment effects observed in resource-rich settings. From an equity and accessibility perspective, the high cost of virtual reality equipment, limited digital infrastructure, and shortages of trained mental health providers may restrict the feasibility of VRET in many low- and middle-income countries. These structural barriers highlight important gaps between demonstrated efficacy and real-world applicability across diverse health systems. Although emerging low-cost and smartphone based virtual reality platforms may reduce some access barriers, their clinical effectiveness and implementation feasibility in resource-constrained settings remain insufficiently evaluated. Consequently, the global relevance of VRET should be interpreted cautiously.

### Strengths and limitations

This study has several strengths. First, to the best of our knowledge, this meta-analysis provides the most methodologically rigorous synthesis to date of randomised controlled trial evidence on the effects of VRET in clinically diagnosed anxiety-related disorders. By restricting inclusion to randomised controlled trials and clinically confirmed diagnoses, this study enhances internal validity and strengthens causal inference compared with previous syntheses that pooled heterogeneous study designs or nonclinical populations. Second, this study was adopted a transdiagnostic analytical framework to examine shared mechanisms of fear acquisition, avoidance, and extinction across anxiety-related disorders. Rather than assuming homogeneity, diagnostic categories and intervention characteristics were examined through predefined subgroup and moderator analyses, allowing heterogeneity in mechanisms, outcome measures, and effect sizes to be evaluated transparently. Third, we conducted a comprehensive systematic search without language restrictions and adhered to the PRISMA guidelines. The study protocol was prospectively registered to enhance transparency and reduce the risk of selective reporting. Collectively, these strengths contribute to a robust and clinically relevant evidence base that informs both evidence-based practice and digital mental health implementation.

Despite these strengths, several limitations acknowledged. First, although a transdiagnostic approach is conceptually justified for exposure-based interventions, anxiety-related disorders differ in symptom structure, outcome measurement, and expected magnitude of treatment effects. Consequently, pooled estimates should be interpreted as reflecting average effects across disorder-specific efficacy. Second, the included studies were conducted predominantly in high-income countries, reflecting where VRET has most frequently been developed and implemented. This geographic concentration limits the external validity and immediate policy relevance of the findings for low- and middle-income countries, where disparities in mental health infrastructure, technological access, and workforce capacity may affect feasibility and scalability. Third, heterogeneity remained moderate to high in several analyses, indicating residual variability that could not be fully explained despite subgroup and moderator analyses. Fourth, the long-term effects of VRET remain uncertain because most included trials had follow-up durations of six months or less. Fifth, although some studies employed validated psychometric instruments, reliance on subjective-reported may introduce response bias. Finally, publication bias cannot be completely excluded, as studies with nonsignificant findings may be underrepresented despite the use of funnel plot inspection and statistical tests. Future research should prioritise disorder-specific randomized trials conducted in diverse economic settings, with longer follow-up periods and greater standardisation of outcome measures, to strengthen the evidence base and inform equitable global implementation.

## CONCLUSIONS

In the evolving field of psychotherapy, VR technology has been increasingly investigated as an adjunct to exposure-based interventions, particularly for anxiety related disorders. This meta-analysis demonstrates that VRET is associated with meaningful reductions in anxiety, phobia, and PTSD, as well as improvements in related behavioural outcomes. These findings support the clinical value of VRET as an innovative exposure-based therapeutic approach within controlled treatment settings. However, the current evidence base is derived predominantly from studies conducted in high income countries and further research conducted in low- and middle-income countries is necessary to evaluate its equity, feasibility, and scalability before broader global implementation can be supported. In addition, uncertainty remains regarding the durability of treatment effects, as many included trials had relatively short follow up periods. For health care professionals, these findings underscore the potential of VRET as a promising therapeutic tool, while highlighting the importance of appropriate training, thoughtful integration into clinical practice, and collaboration with technology developers to optimise usability and adherence. Future randomised controlled trials incorporating longer follow up, implementation outcomes, and diverse economic settings will be essential to strengthen the evidence base and inform equitable and sustainable adoption of VRET in global mental health care.

## Additional material


Online Supplementary Document
