# Supplementary material for: Innovative virtual reality exposure therapy for anxiety and posttraumatic stress disorder: a meta-analysis of randomised controlled trials
Source: J Glob Health. 2026 Mar 27;16:04090. doi: 10.7189/jogh.16.04090 (PMC13033945; doi:10.7189/jogh.16.04090)

Supplement to:Chang YC, Arifin H, Hung TM, Lin CL, Ye JY, Pien LC, Su PY, Chen R, Chou KR. Innovative virtual reality exposure therapy for anxiety and post-traumatic stress disorder: a meta-analysis of randomised controlled trials. J Glob Health. 2026;16:04090.

**Supplementary list**	Page Number
**Table S1.** Search Terms.	1
**Table S2.** Syntaxes.	4
**Table S3.** Sensitivity analyses.	9
**Table S4.** Publication bias.	10
**Figure S1.** Risk of bias assessment.	11
**Figure S2.** Funnel plot on anxiety.	12
**Figure S3.** Funnel plot on phobia.	13

Table S1. Search Terms.

Databases	Population	Intervention	Comparison	Outcome
**CINAHL** (MH exact Subject Headings)	- Anxiety (MH)<br>- "Post-Traumatic Stress Disorder"<br>- "Stress Disorders, Post-Traumatic" (MH)<br>- PTSD<br>- "Panic disorder" (MH)<br>- Panic<br>- "Phobic disorders" (MH)<br>- Phobia	- "Virtual Reality"<br>- "Virtual Reality Exposure Therapy"<br>- "Augmented Reality"<br>- "Mixed Reality"<br>- "Implosive Therapies"<br>- "Flooding Therapy"<br>- "Exposure Therapy"	- no intervention<br>- usual care<br>- wait-list	- Anxiety (MH)<br>- "Post-Traumatic Stress Disorder"<br>- "Stress Disorders, Post-Traumatic" (MH)<br>- PTSD<br>- "Panic disorder" (MH)<br>- "Phobic disorders" (MH)<br>- Phobia
**Cochrane**	- Anxiety (MeSH)<br>- "Post-Traumatic Stress Disorder"<br>- "Stress Disorders, Post-Traumatic" (MeSH)<br>- PTSD<br>- Panic (MeSH)<br>- "Phobic disorders" (MeSH)<br>- Phobia	- "Virtual Reality" (MeSH)<br>- "Virtual Reality Exposure Therapy" (MeSH)<br>- "Augmented Reality" (MeSH)<br>- "Mixed Reality"<br>- "Implosive Therapies" (MeSH)<br>- "Flooding Therapy"<br>- "Exposure Therapy"	- no intervention<br>- usual care<br>- wait-list	- Anxiety (MeSH)<br>- "Post-Traumatic Stress Disorder"<br>- "Stress Disorders, Post-Traumatic" (MeSH)<br>- PTSD<br>- Panic (MeSH)<br>- "Phobic disorders" (MeSH)<br>- Phobia
**Embase** (Emtree Terms)	- Anxiety (EM)<br>- "Post-Traumatic Stress Disorder"<br>- "posttraumatic stress disorder" (EM)<br>- PTSD<br>- Panic (EM)<br>- Phobia (EM)	- "Virtual Reality" (EM)<br>- "Virtual Reality Exposure Therapy" (EM)<br>- "Augmented Reality" (EM)<br>- "Mixed Reality"	- no intervention<br>- usual care<br>- wait-list	- Anxiety (EM)<br>- "Post-Traumatic Stress Disorder"<br>- "posttraumatic stress disorder" (EM)<br>- PTSD<br>- Panic (EM)<br>- Phobia (EM)

Databases	Population	Intervention	Comparison	Outcome
		- "Implosive Therapies" (EM)<br>- "Flooding Therapy"<br>- "Exposure Therapy" (EM)		
**Medline**	- Anxiety (MeSH)<br>- "Post-Traumatic Stress Disorder"<br>- "Stress Disorders, Post-Traumatic" (MeSH)<br>- PTSD<br>- Panic (MeSH)<br>- "Phobic disorders" (MeSH)<br>- Phobia	- "Virtual Reality" (MeSH)<br>- "Virtual Reality Exposure Therapy" (MeSH)<br>- "Augmented Reality" (MeSH)<br>- "Mixed Reality"<br>- "Implosive Therapies" (MeSH)<br>- "Flooding Therapy"<br>- "Exposure Therapy"	- no intervention<br>- usual care<br>- wait-list	- Anxiety (MeSH)<br>- "Post-Traumatic Stress Disorder"<br>- "Stress Disorders, Post-Traumatic" (MeSH)<br>- PTSD<br>- Panic (MeSH)<br>- "Phobic disorders" (MeSH)<br>- Phobia
**PsycINFO** (Mesh Subject Headings)	- Anxiety (MA)<br>- "Post-Traumatic Stress Disorder"<br>- PTSD<br>- Panic (MA)<br>- "Phobic disorders" (MA)<br>- Phobia	- "Virtual Reality" (MeSH)<br>- "Virtual Reality Exposure Therapy" (MeSH)<br>- "Augmented Reality" (MeSH)<br>- "Mixed Reality"<br>- "Implosive Therapies" (MeSH)<br>- "Flooding Therapy"<br>- "Exposure Therapy"	- no intervention<br>- usual care<br>- wait-list	- Anxiety (MeSH)<br>- "Post-Traumatic Stress Disorder"<br>- "Stress Disorders, Post-Traumatic" (MeSH)<br>- PTSD<br>- Panic (MeSH)<br>- "Phobic disorders" (MeSH)<br>- Phobia<br>-

Databases	Population	Intervention	Comparison	Outcome
**PubMed** (Medical Subject Headings)	- Anxiety (MeSH)<br>- "Post-Traumatic Stress Disorder"<br>- "Stress Disorders, Post-Traumatic" (MeSH)<br>- PTSD<br>- Panic (MeSH)<br>- "Phobic disorders" (MeSH)<br>- Phobia	- "Virtual Reality" (MeSH)<br>- "Virtual Reality Exposure Therapy" (MeSH)<br>- "Augmented Reality" (MeSH)<br>- "Mixed Reality"<br>- "Implosive Therapies" (MeSH)<br>- "Flooding Therapy"<br>- "Exposure Therapy"	- no intervention<br>- usual care<br>- wait-list	- Anxiety (MeSH)<br>- "Post-Traumatic Stress Disorder"<br>- "Stress Disorders, Post-Traumatic" (MeSH)<br>- PTSD<br>- Panic (MeSH)<br>- "Phobic disorders" (MeSH)<br>- Phobia
**Web of Science**	- Anxiety (MeSH)<br>- "Post-Traumatic Stress Disorder"<br>- "Stress Disorders, Post-Traumatic" (MeSH)<br>- PTSD<br>- Panic (MeSH)<br>- "Phobic disorders" (MeSH)<br>- Phobia	- "Virtual Reality" (MeSH)<br>- "Virtual Reality Exposure Therapy" (MeSH)<br>- "Augmented Reality" (MeSH)<br>- "Mixed Reality"<br>- "Implosive Therapies" (MeSH)<br>- "Flooding Therapy"<br>- "Exposure Therapy"	- no intervention<br>- usual care<br>- wait-list	- Anxiety (MeSH)<br>- "Post-Traumatic Stress Disorder"<br>- "Stress Disorders, Post-Traumatic" (MeSH)<br>- PTSD<br>- Panic (MeSH)<br>- "Phobic disorders" (MeSH)<br>- Phobia

Table S2. Syntaxes

Databases	Syntaxes	Number	Total
**CINAHL**	S1: MH Anxiety OR TI Anxiety OR AB Anxiety OR MH “Stress Disorders, Post-Traumatic” OR TI “Stress Disorders, Post-Traumatic” OR AB “Stress Disorders, Post-Traumatic” OR TI “Post-Traumatic Stress Disorder” OR AB “Post-Traumatic Stress Disorder” OR TI PTSD OR AB PTSD OR MH “Panic disorder” OR TI “Panic disorder” OR AB “Panic disorder” OR TI Panic OR AB Panic OR MH “Phobic disorders” OR TI “Phobic disorders” OR AB “Phobic disorders” OR TI Pania OR AB Pania	165,715	41
	S2: TI “Virtual Reality” OR AB “Virtual Reality” OR TI “Virtual Reality Exposure Therapy” OR AB “Virtual Reality Exposure Therapy” OR TI “Augmented Reality” OR AB “Augmented Reality” OR TI “Mixed Reality” OR AB “Mixed Reality”	8,419	
	S3: TI “Implosive Therapies” OR AB “Implosive Therapies” OR TI “Flooding Therapy” OR AB “Flooding Therapy” OR TI “Exposure Therapy” OR AB “Exposure Therapy”	798	
	S4: S2 AND S3	152	
	S5: MH Randomized Controlled Trials OR TI Randomized Controlled Trials OR AB Randomized Controlled Trials OR TI Randomised Controlled Trials OR AB Randomised Controlled Trials OR TI Randomized OR AB Randomized OR TI Randomised OR AB Randomised OR TI Randomization OR AB Randomization OR TI Randomisation OR AB Randomisation OR TI randomly OR AB randomly	379,388	
	S6: S1AND S4 AND S5	41	
**Cochrane**	#1: MeSH descriptor: [Anxiety] explode all trees	13,026	398
	#2: (Post-Traumatic Stress Disorder)	5,648	
	#3: MeSH descriptor: [Stress Disorders, Post-Traumatic] explode all trees	4,291	
	#4: (PTSD)	6,722	
	#5: MeSH descriptor: [Panic] explode all trees	286	
	#6: MeSH descriptor: [Phobic Disorders] explode all trees	2,173	
	#7: (Phobia)	3,160	
	#8: (Anxiety OR Panic OR (Phobic Disorders))	88,219	
	#9: #1 OR #2 OR #3 OR #4 OR #5 OR #6 OR #7 OR #8	94,529	
	#10: MeSH descriptor: [Virtual Reality] explode all trees	1,343	

Databases	Syntaxes	Number	Total
	#11: MeSH descriptor: [Virtual Reality Exposure Therapy] explode all trees	361	
	#12: MeSH descriptor: [Augmented Reality] explode all trees	125	
	#13: ((Virtual Reality) OR (Virtual Reality Exposure Therapy) OR (Augmented Reality) OR (Mixed Reality))	9,149	
	#14: #10 OR #11 OR #12 OR #13	9,162	
	#15: MeSH descriptor: [Implosive Therapy] explode all trees	718	
	#16: ((Implosive Therapy) OR (Flooding Therapy) OR (Exposure Therapy))	33,030	
	#17: #15 OR #16	33,030	
	#18: #14 AND #17	3,022	
	#19: MeSH descriptor: [Randomized Controlled Trial] explode all trees	34	
	#20: MeSH descriptor: [Random Allocation] explode all trees	25,549	
	#21: ((Randomized Controlled Trial) OR (Randomised Controlled Trials) OR Randomized OR Randomised OR Randomization OR Randomisation OR randomly)	1,435,673	
	#22: #19 OR #20 OR #21	1,442,559	
	#23: #3 AND #4 AND #9 AND #13	507	
	#24: Trials matching	398	
**Embase**	#1: Anxiety:ab,ti OR Anxiety/exp OR ‘posttraumatic stress disorder’/exp OR ‘posttraumatic stress disorder’:ab,ti OR ‘Post-Traumatic Stress Disorder’:ab,ti OR PTSD:ab,ti OR Panic:ab,ti OR Panic/exp OR Phobia:ab,ti OR Phobia/exp	618,104	313
	#2: “Virtual Reality”:ab,ti OR “Virtual Reality”/exp OR “Virtual Reality Exposure Therapy”:ab,ti OR “Virtual Reality Exposure Therapy”/exp OR “Augmented Reality”:ab,ti OR “Augmented Reality”/exp OR “Mixed Reality”:ab,ti	44,010	
	#3: “Implosive Therapies”/exp OR “Implosive Therapies”:ab,ti OR “Exposure Therapy”/exp OR “Exposure Therapy”:ab,ti OR “Flooding Therapy”:ab,ti	5,413	
	#4: #2 AND #3	1,504	
	#5: 'randomized controlled trial'/exp OR 'randomized controlled trial':ab,ti OR 'randomised controlled trial':ab,ti OR randomized:ab,ti OR randomised:ab,ti OR 'randomization'/exp OR randomization:ab,ti OR randomisation:ab,ti OR randomly:ab,ti	1,928,658	
	#1 AND #4 AND #5	313	

Databases	Syntaxes	Number	Total
**Medline**	1: exp Anxiety/ or Anxiety.mp. or (exp Stress Disorders, Post-Traumatic/ or Stress Disorders, Post-Traumatic.mp.) or (exp Panic/ or Panic.mp.) or (exp Phobic Disorders/ or Phobic Disorders.mp.) or "Post-Traumatic Stress Disorder".mp. or PTSD.mp. or Phobia.mp.	413,600	302
	2: exp Virtual Reality/ or Virtual Reality.mp. or (exp Virtual Reality Exposure Therapy/ or Virtual Reality Exposure Therapy.mp.) or (exp Augmented Reality/ or Augmented Reality.mp.) or Mixed Reality.mp.	29,150	
	3: exp Implosive Therapy/ or Implosive Therapy.mp. or Flooding Therapy.mp. or Exposure Therapy.mp.	4,591	
	4: 2 AND 3	1,571	
	5: "randomized controlled trial".mp. or Randomized Controlled Trial/	687,996	
	6: "randomised controlled trial".mp.	36,421	
	7: randomized.mp.	1,120,846	
	8: randomised.mp.	146,486	
	9: randomization.mp. or Random Allocation/	159,362	
	10: randomisation.mp.	13,819	
	11: randomly.mp.	455,976	
	12: 5 OR 6 OR 7 OR 8 OR 9 OR 10 OR 11	1,520,311	
	1 AND 4 AND 12	302	
**PsycINFO**	S1: MA Anxiety OR TI Anxiety OR AB Anxiety OR “Post-Traumatic Stress Disorder” OR TI “Post-Traumatic Stress Disorder” OR AB “Post-Traumatic Stress Disorder” OR TI PTSD OR AB PTSD OR MA Panic OR TI Panic OR AB Panic OR MA “Phobic disorders” OR TI “Phobic disorders” OR AB “Phobic disorders” OR TI Phobia OR AB Phobia	702,496	75
	S2: MA “Virtual Reality” OR TI “Virtual Reality” OR AB “Virtual Reality” OR MA “Virtual Reality Exposure Therapy” OR TI “Virtual Reality Exposure Therapy” OR AB “Virtual Reality Exposure Therapy” OR MA “Augmented Reality” OR TI “Augmented Reality” OR AB “Augmented Reality” OR TI “Mixed Reality” OR AB “Mixed Reality”	1,535	
	S3: MA “Implosive Therapies” OR TI “Implosive Therapies” OR AB “Implosive Therapies” OR TI “Flooding Therapy” OR AB	897	

Databases	Syntaxes	Number	Total
	"Flooding Therapy" OR TI "Exposure Therapy" OR AB Exposure Therapy"		
	S4: S2 AND S3	2,418	
	S5: MA "randomized controlled trial" OR TI "randomized controlled trial" OR AB "randomized controlled trial" OR TI "randomised controlled trial" OR AB "randomised controlled trial" OR TI randomized OR AB randomized OR TI randomised OR AB randomised OR TI randomization OR AB Randomization	111,175	
	S6: TI Randomisation OR AB Randomisation OR TI randomly OR AB randomly	91,332	
	S7: S5 OR S6	182,510	
	S1 AND S4 and S7	75	
**PubMed**	#1: Search: (((((((("anxiety"[MeSH Terms]) OR (Anxiety[Title/Abstract])) OR ("Stress Disorders, Post-Traumatic"[MeSH Terms])) OR ("Post-Traumatic Stress Disorder"[Title/Abstract])) OR (PTSD[Title/Abstract])) OR (Panic[MeSH Terms])) OR (Panic[Title/Abstract])) OR ("Phobic disorders"[MeSH Terms])) OR ("Phobic disorders"[Title/Abstract])) OR (Phobia[Title/Abstract])	393,784	169
	#2: "Virtual Reality"[MeSH Terms] OR "Virtual Reality"[Title/Abstract] OR "Virtual Reality Exposure Therapy"[MeSH Terms] OR "Virtual Reality Exposure Therapy"[Title/Abstract] OR "Augmented Reality"[MeSH Terms] OR "Augmented Reality"[Title/Abstract] OR "Mixed Reality"[Title/Abstract] Sort by: Most Recent	29,093	
	#3: "Implosive Therapies"[MeSH Terms] OR "Implosive Therapies"[Title/Abstract] OR "Flooding Therapy"[Title/Abstract] OR "Exposure Therapy"[Title/Abstract]	2,731	
	#4: #2 AND #3	653	
	#5: ((((((("randomized controlled trial"[Title/Abstract])) OR ("randomised controlled trial"[Title/Abstract])) OR (randomized[Title/Abstract])) OR (randomised[Title/Abstract])) OR (randomization[Title/Abstract])) OR (randomisation[Title/Abstract])) OR (randomly[Title/Abstract]))	1,260,231	
	#6: #1 AND #4 AND #5	169	
**Web of Science**	1: ((((((((((TI=(Anxiety)) OR AB=(Anxiety)) OR TI=("Post-Traumatic Stress Disorder")) OR AB=("Post-Traumatic Stress	371,189	143

Databases	Syntaxes	Number	Total
	Disorder”)) OR TI=(PTSD)) OR AB=(PTSD)) OR TI=(Panic)) OR AB=(Panic)) OR TI=(“Phobic disorders”)) OR AB=(“Phobic disorders”)) OR TI=(Phobia)) OR AB=(Phobia)		
	2: (((((((TI=(“Virtual Reality”)) OR AB=(“Virtual Reality”)) OR TI=(“Virtual Reality Exposure Therapy”)) OR AB=(“Virtual Reality Exposure Therapy”)) OR TI=(“Augmented Reality”)) OR AB=(“Augmented Reality”)) OR TI=(“Mixed Reality”)) OR AB=(“Mixed Reality”)	44,733	
	3: “Implosive Therapies” (Title) OR “Implosive Therapies” (Abstract) OR “Flooding Therapy” (Title) OR “Flooding Therapy” (Abstract) OR “Exposure Therapy” (Title) OR “Exposure Therapy” (Abstract)	2,634	
	4: #2 AND #3	542	
	5: (((((((((((((TI=("randomized controlled trial")) OR AB=("randomized controlled trial")) OR TI=("randomised controlled trial")) OR AB=("randomised controlled trial")) OR TI=(randomized)) OR AB=(randomized)) OR TI=(randomised)) OR AB=(randomised)) OR TI=(randomization)) OR AB=(randomization)) OR TI=(randomisation)) OR AB=(randomisation) OR TI=(randomly)) OR AB=(randomly))	1,372,166	
	5: #1 AND #4 AND #5	143	
		**TOTAL**	**1,441**

Table S3. Sensitivity analyses.

Outcomes	Number of studies	Sensitivity	I^2	Hedge's g	95% Confidence interval	P-value
Anxiety						
Post-intervention effects	13	Remove one study	60.867%	-0.613	-0.896, -0.329	<0.001
Phobia						
Post-intervention effects	11	Remove one study	78.914%	-1.000	-1.390, -0.611	<0.001
	10	Remove one high risk study	78.574%	-0.918	-1.303, -0.533	<0.001
PTSD						
Post-intervention effects	7	Remove one study	<0.001%	-0.518	-0.733, -0.303	<0.001
Behavioural (Approach) Post-intervention effects	4	Remove one study	59.700%	0.631	0.112, 1.150	0.017
Behavioural (Avoidance) Post-intervention effects	3	Remove one study	90.949%	-1.335	-2.665, -0.004	0.049

Table S4. Publication bias

Outcomes	Number of studies	Studies trimmed	Observed value		Adjusted values		Egger's test
			Hedges' g	95% Confidence interval	Hedges' g	95% Confidence interval	*P-value*
Anxiety							
Post-intervention effects	13	2	-0.613	-0.896, -0.329	-0.708	-0.996, -0.420	0.025
Phobia							
Post-intervention effects	10	0	-0.982	-1.365, -0.600			0.602

Figure S1. Risk of bias assessment

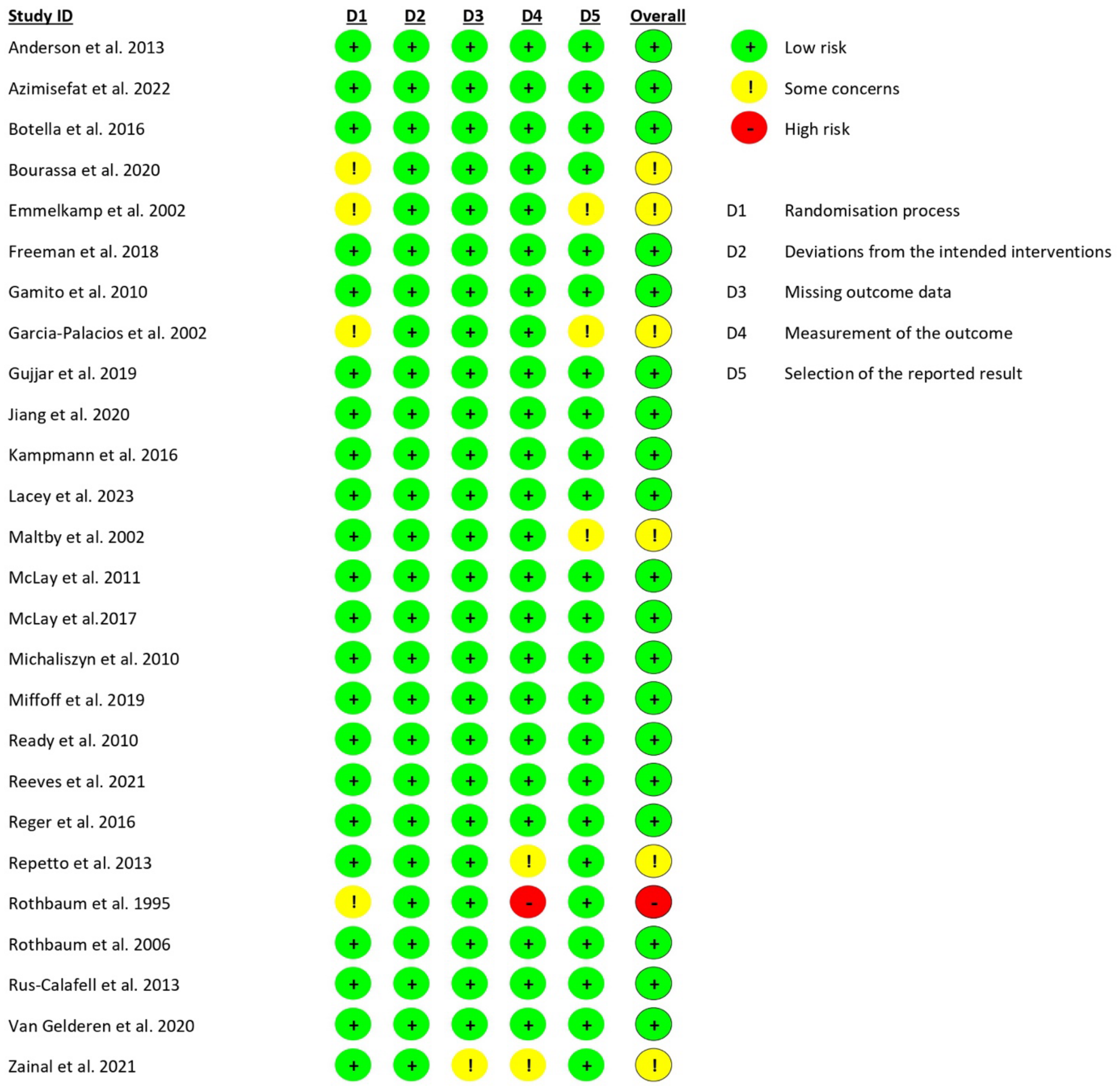

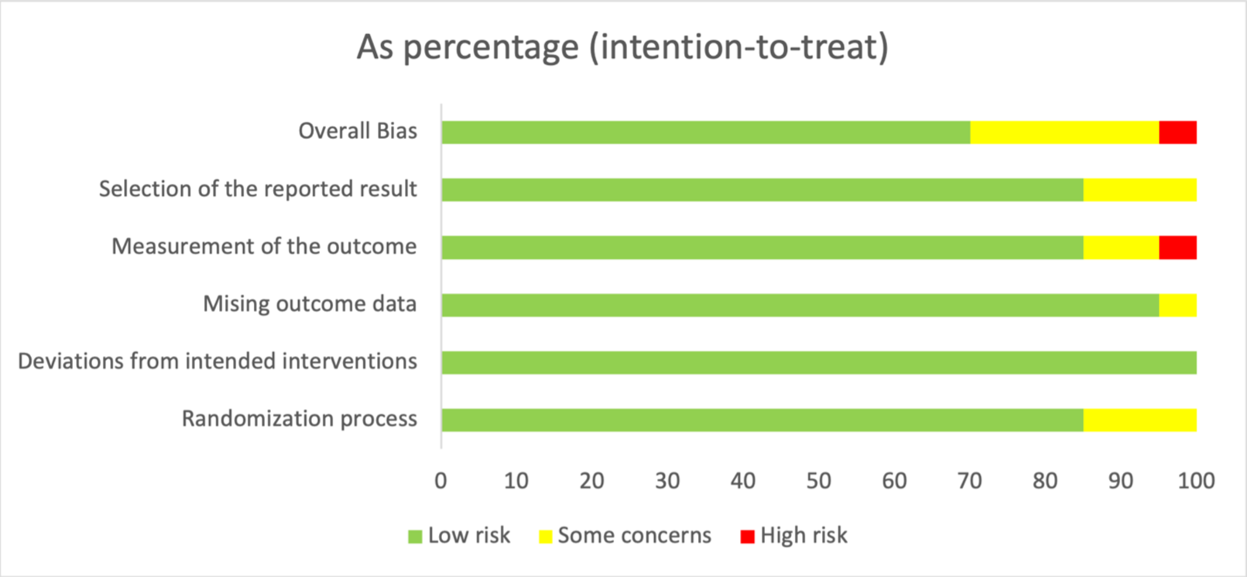

Figure S2. Funnel plot on Anxiety.

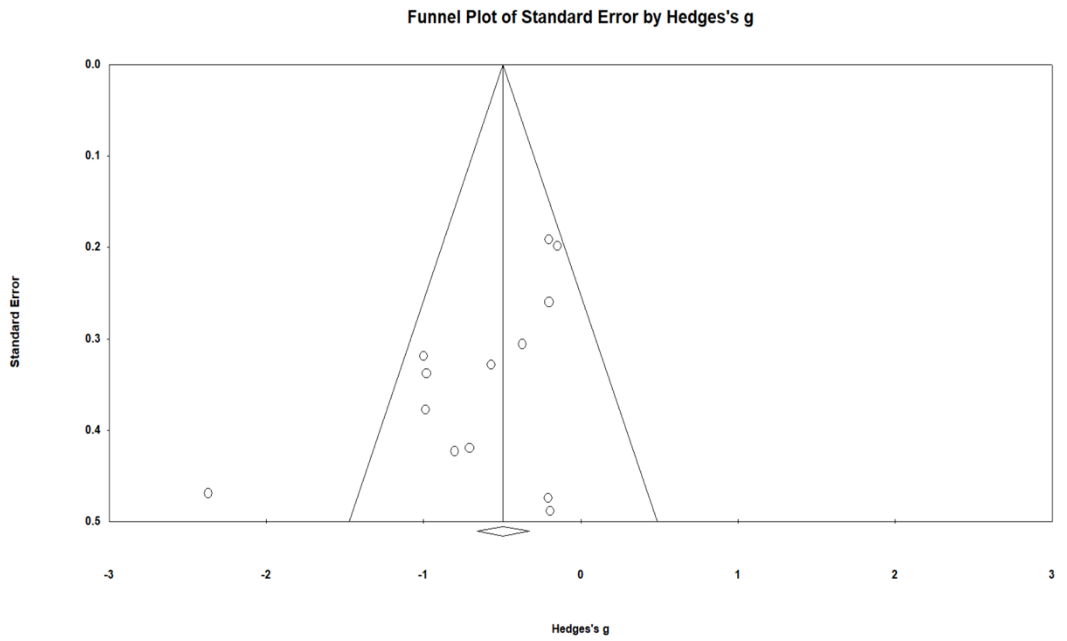

Figure S3. Funnel plot on Phobia.

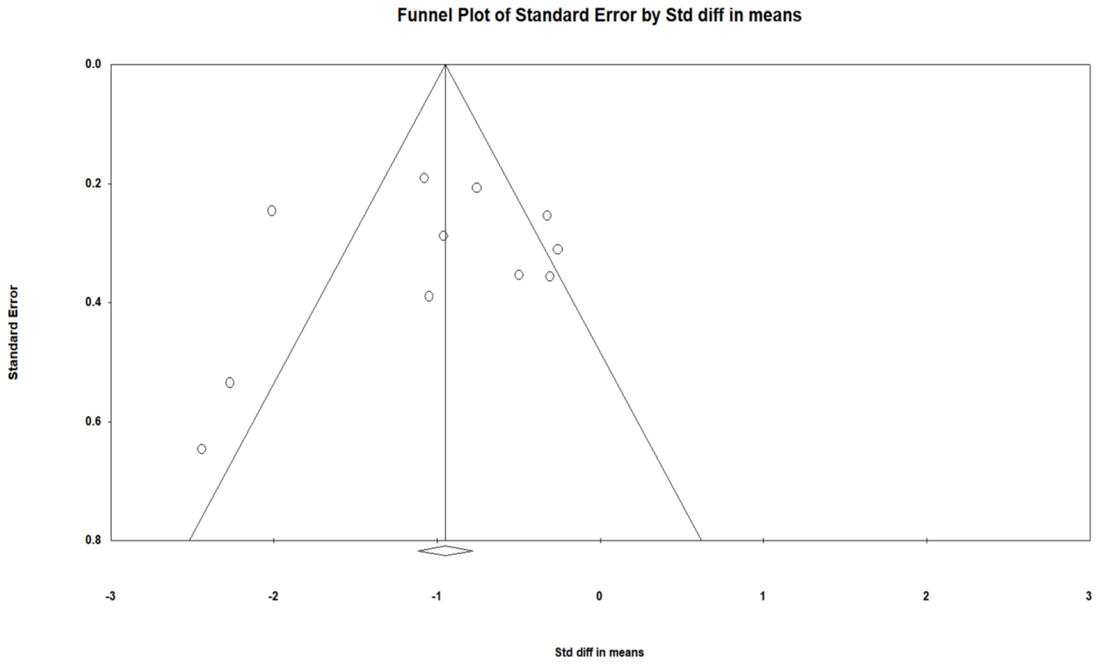

Supplement: Online Supplementary Document [file jogh-16-04090-s001.pdf]
